# Supplementary material for: A protocol for a multi-site cohort study to evaluate child and adolescent mental health service transformation in England using the i-THRIVE model
Source: PLoS One. 2023 May 8;18(5):e0265782. doi: 10.1371/journal.pone.0265782 (PMC10166497; doi:10.1371/journal.pone.0265782)
Supplement: S1 Fig — A figure and accompanying text providing a description of the THRIVE framework. (PDF) [file pone.0265782.s001.pdf]

*Fig. 1. The THRIVE Framework needs-based categories and corresponding inputs (figure from Wolpert et al 2019)*

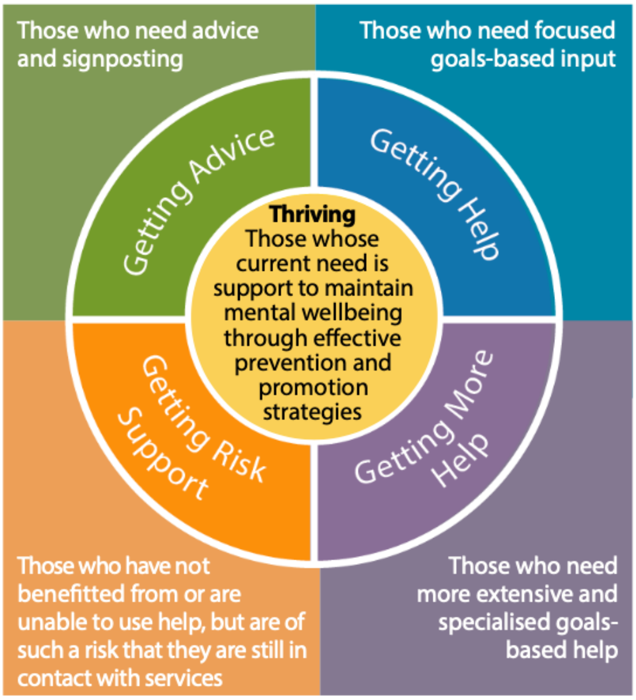


The THRIVE Framework is a novel theoretical model for service delivery that was developed by the Anna Freud National Centre for Children and Families and the Tavistock and Portman NHS Foundation Trust. THRIVE proposes a set of principles around how care services should be organised that offers an integrated, person-centred, and needs-led approach to delivering mental health services for children and young people (CYP) and their families (Wolpert et al, 2014). This includes actively involving CYP and their families in decisions and choices about the support they receive. These principles also acknowledge the limitation of evidence-based interventions and the resources available, and distinguishes between intervention (or 'treatment') and risk support. A greater emphasis is also placed on supporting young people through communities to build strength. Unlike the existing tiered model of CAMHS, the THRIVE model proposes a care system that is delivered according to an individuals' needs, rather than by severity or diagnosis. Need is conceptualised in the model according to five categories: Getting Advice and Signposting, Getting Help, Getting More Help, Getting Risk

Support, and Thriving. The first four categories relate to the needs and preferences of CYP experiencing mental health problems, while ‘Thriving’ represents a preventative mental health and wellbeing approach relevant to all CYP. (See Wolpert et al., 2014, 2015, 2019 for further details).
