# Supplementary figures and images for: A protocol for a multi-site cohort study to evaluate child and adolescent mental health service transformation in England using the i-THRIVE model
Source: PLoS One. 2023 May 8;18(5):e0265782. doi: 10.1371/journal.pone.0265782 (PMC10166497; doi:10.1371/journal.pone.0265782)

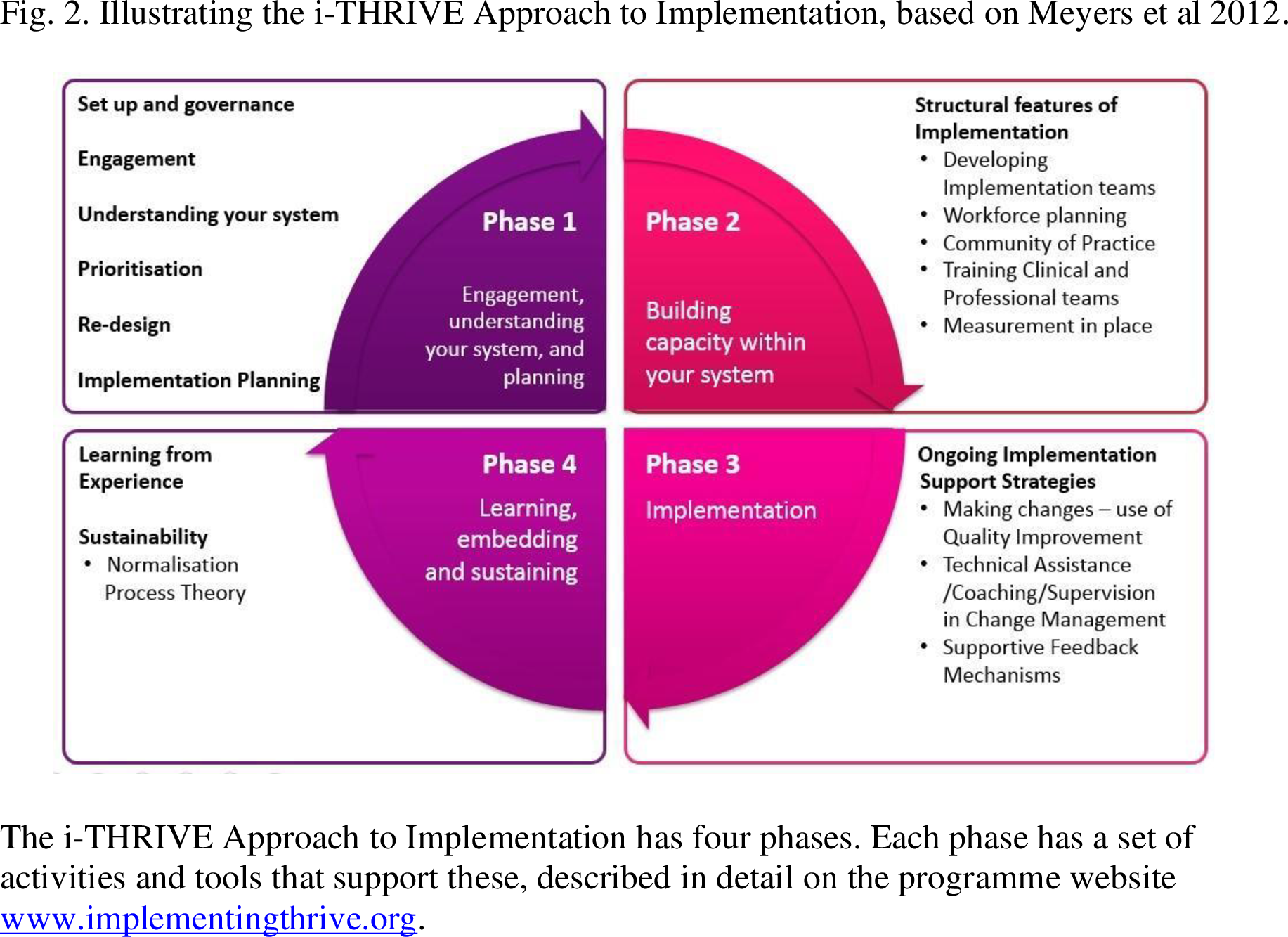

Supplement: S2 Fig — A figure illustrating the i-THRIVE Approach to illustration together with the details of where to find additional information on the website. (TIF) [file pone.0265782.s002.tif]

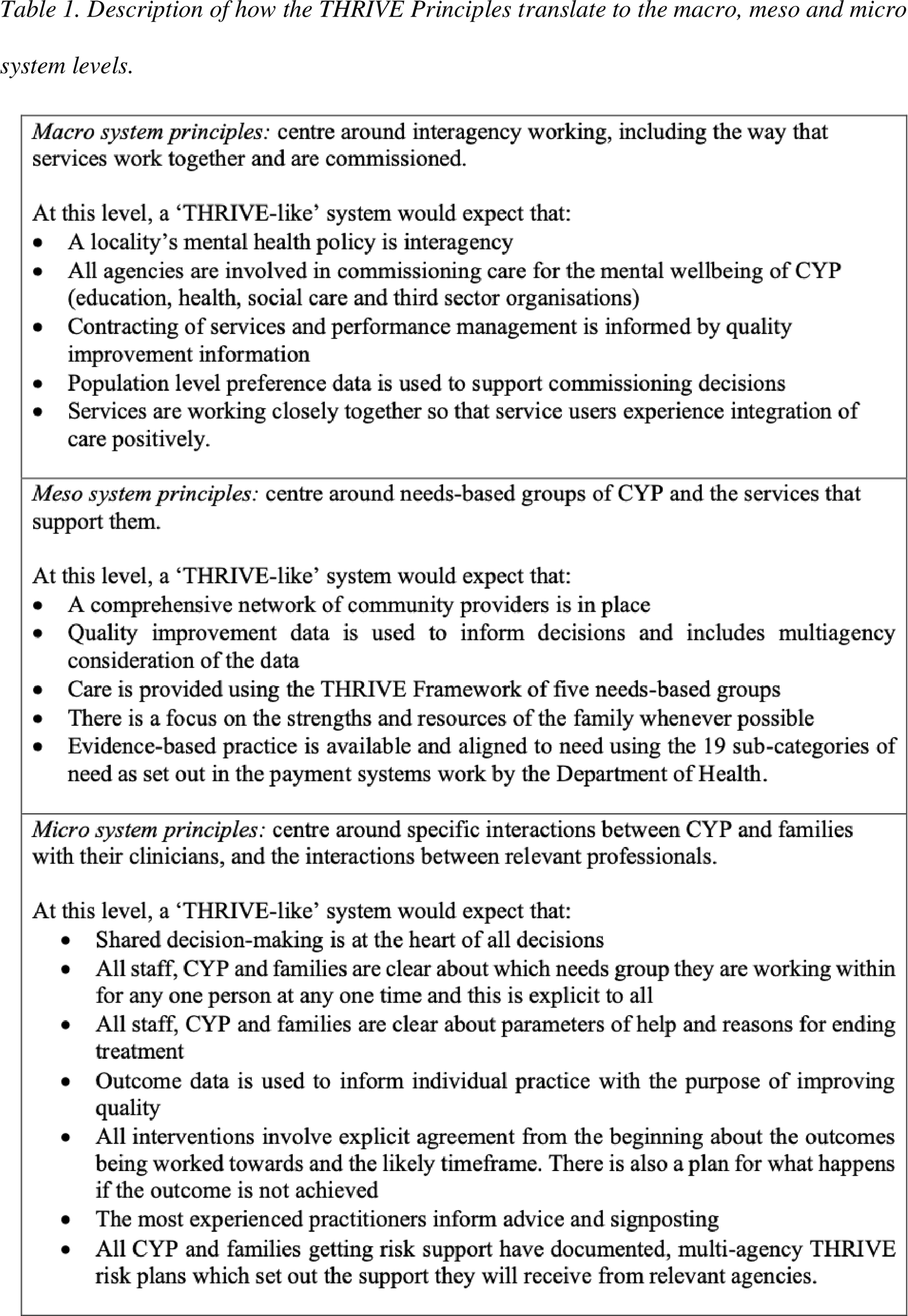

Supplement: S1 Table — A table that describes the THRIVE principles for each of the three system levels. (TIF) [file pone.0265782.s004.tif]

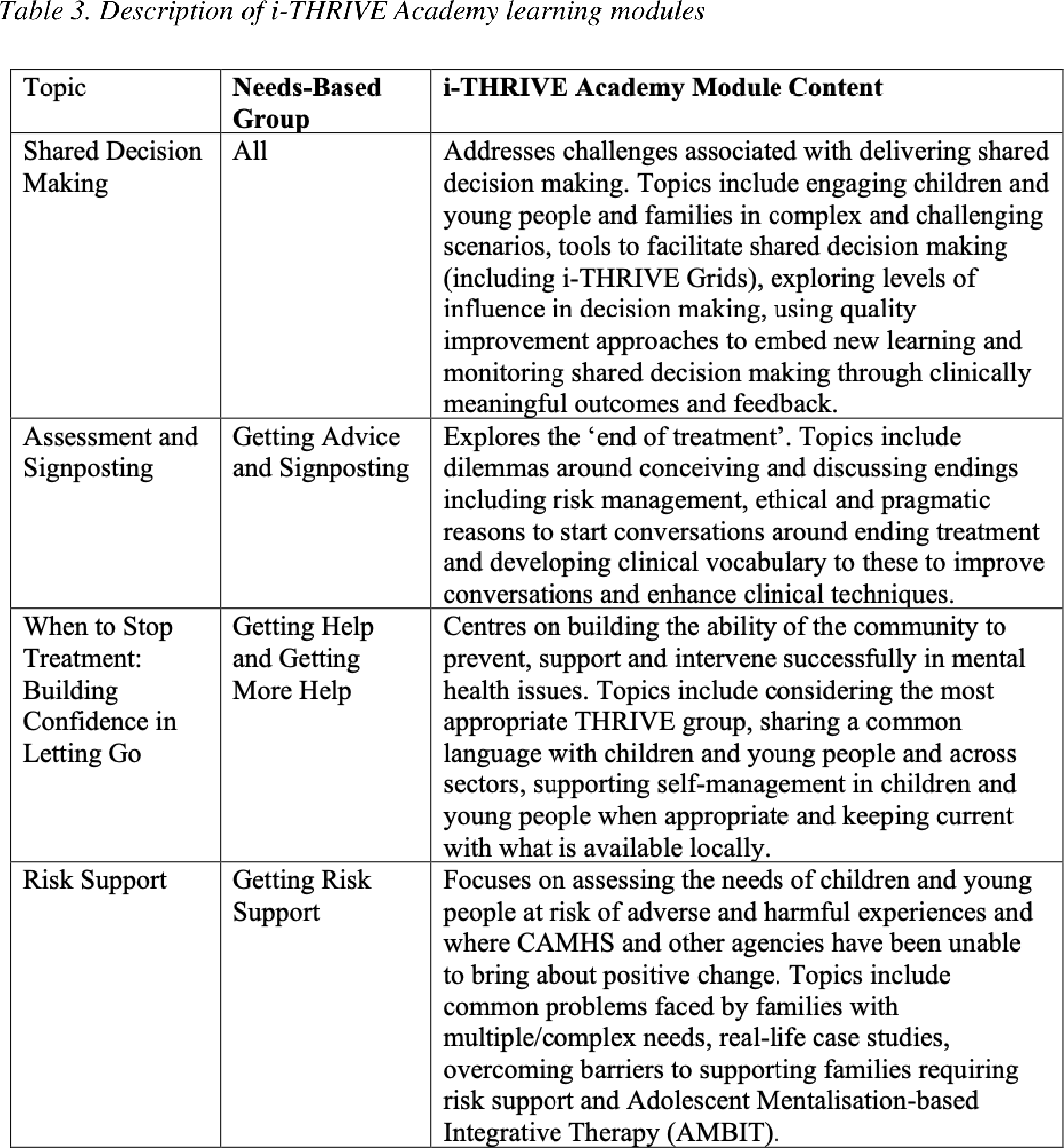

Supplement: S2 Table — A table describing each of the four i-THRIVE Academy Modules, and how they relate to the THRIVE framework. (TIF) [file pone.0265782.s005.tif]

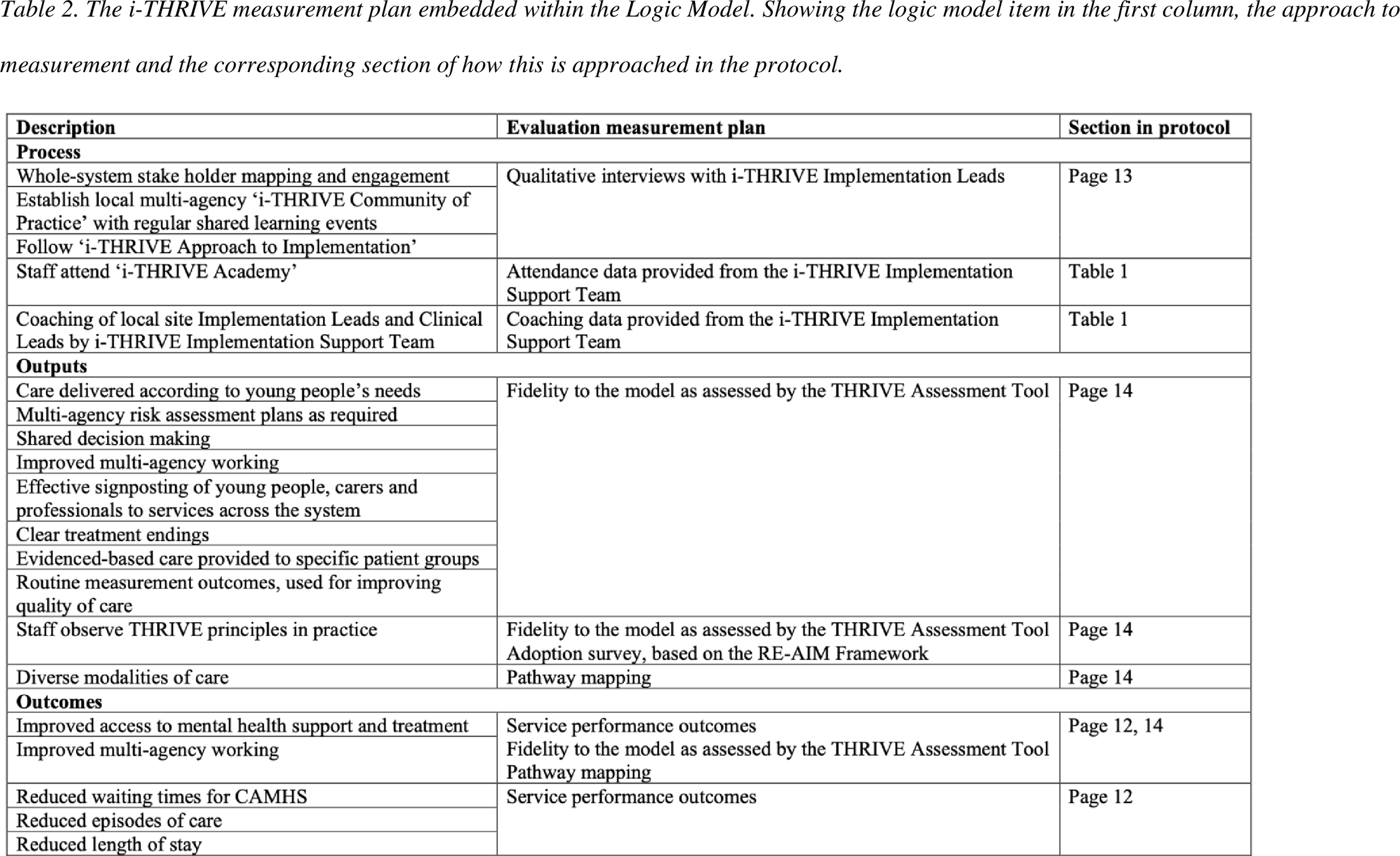

Supplement: S3 Table — Showing the logic model item in the first column, the approach to measurement and the corresponding section of how this is approached in the protocol. A table showing how each aspect of the logic model has been translated into an outcome measure, and where the description can be found in the protocol. (TIF) [file pone.0265782.s006.tif]
