# Supplementary material for: A protocol for a multi-site cohort study to evaluate child and adolescent mental health service transformation in England using the i-THRIVE model
Source: PLoS One. 2023 May 8;18(5):e0265782. doi: 10.1371/journal.pone.0265782 (PMC10166497; doi:10.1371/journal.pone.0265782)
Supplement: S3 Fig — A figure of the Thrive assessment tool that will be used to measure the fidelity in each of the sites. (PDF) [file pone.0265782.s003.pdf]

Collaboration for Leadership in
Applied Health Research and Care
North Thames

i-THRIVE Implementation Assessment Tool Rating Sheet: Macro System Level

THRIVE Principle<br><br>A locality's mental health policy is interagency	1	2	3	4	Score
**Ma 1b: Policy involving MH**	No policy on how a locality will deliver improved outcomes for CYP mental health.	There is a policy on how to improve children's MH, however this does not cover all aspects of care nor does it involve the whole system.	There is a policy on how to improve MH for CYP, however it either does not cover all aspects of care, or does not cover the whole system	There is a policy statement/ document that clearly articulates the locality's approach to delivering improved outcomes for children and young people's mental health, including details for each sector.	
**Ma 1c: Jointly created policy**	No evidence of any joint working between agencies.	Some evidence of joint working between agencies, but this is limited to two different agencies (health, education, social care or education).	Creation of policy has involved at least three different agencies (health, education, social care or third sector).	There is a policy statement/ document which has been jointly created between health, care, education and third sector input.	
**Ma 1d: Child health in STP and LTP**	Child mental health is not included in the Sustainability and Transformation Plans (STPs) or Local Transformation Plans (LTPs).	Child mental health is included in either the LTP or STP, although this is minimal.	Child mental health is included in both the LTP and STP and there is good strategic coverage for CYP MH, however this is not complete.	Child mental health is included in both the LTP and STP and there are clear strategic plans together with implementation plans that cover CYP MH for the locality.	
**Ma 1e: Implementation plan (includes all agencies)**	There is no implementation plan in place.	There is evidence of an implementation plan, however this is not comprehensive, or it does not include other agencies.	There is a clear implementation plan in place, however this does not span all agencies in the locality. There is evidence that the plan is not being delivered in services as yet.	There is a clear implementation plan in place spanning all agencies in the locality. There is evidence that this is being delivered 'on the ground'.	

THRIVE Principle<br><br>All agencies are involved in commissioning care for the mental health and wellbeing of CYP (education, health, social care, third sector)	1	2	3	4	Score
**Ma 2b: Evidence of joint commissioning**	There are separate commissioning structures for local authority and health.	There is a limited amount of joint commissioning. This may relate to specific projects or services.	There are some forms of joint commissioning. There is a range of established commissioned multia-gency projects, but those collaborations only include two or three agencies, e.g. health care and social care.	Health, local authority, education and the third sector are actively involved in commissioning mental health care for the locality.	
**Ma 2c: Evidence of joint governance structures**	There are no joint governance structures. There are separate governance boards which do no collaborate on development of commissioning plans.	There are separate governance boards that collaborate on the development of their commissioning plans, but no joint governance, strategy or budgeting at the most senior levels of the organisation.	There is a joint governance structure and a joint commissioning board, but this includes only two or three agencies, e.g. health care and social care.	Health, social care, education and the third sector sit within one board and are jointly responsible and accountable for delivery of this strategy and the subsequent outcomes for their population.<br><br>There is a governance structure and all agencies are regular attenders of joint commissioning board meetings.	
**Ma 2d: Joint outcome frameworks**	Each organisation has a separate outcome framework and manages their contracts separately.	There are no jointly owned outcome frameworks, but there is effort to align these and local partners are working towards integration.	Joint outcomes have not been formalised in an outcomes framework but there are agreed objectives which different agencies work towards.	The joint governance body has developed joint outcome frameworks to manage their own performance and to support contracting.	

**Ma 2e: Joint budgets**	There are no joint budgets.	There are joint budgets in some, but not all elements of the localities provision. This may apply to some projects, but not all.	There are joint budgets between two or three agencies, e.g. health and social care, but it does not involve all agencies of the local system.	There are joint budgets in operation across the local system.<br><br>(Example: an effectively functioning devolved system or ACO, with joint governance, strategy, budget, performance framework. The responsibility for delivery of outcomes of the population is jointly owned between agencies).	

THRIVE Principle<br><br>Contracting of services, and the performance management of these, is informed by quality improvement information	1	2	3	4	Score
**Ma 3b: Contract reviews with QI data: regular review of contracts**	There is no regular reviewing of the contracts within a locality.	Commissioners have a schedule in place for reviewing contracts, but this is infrequent, i.e. every few years, or doesn't include all commissioned services.	Commissioners have a schedule in place for reviewing contracts frequently, e.g. once a year, and this covers most/all commissioned services.	Commissioners review contracts regularly (quarterly) and refresh commissioning plans and contracts annually.	
**Ma 3c: Contract reviews with QI data: contract reviews based on quality data**	There is little consideration of performance or quality data during the commissioning cycle.	There is some consideration of data and outcomes in the commissioning cycle.	Data and quality information is often used in developing the contracts, although this may not be systematic.	There are clear agreements about the use of data within contracts and on-going performance management of these. Data is routinely used to inform contracting decisions.	
**Ma 3d: Data systems (+ allows sharing): systems of data collection**	Providers do not have systems in place to report quality data for the management of commissioning contracts.	The systems used by providers which do not allow for easy collection and collation of this data.	The systems used to collate and report data enable data to be provided in a timely manner, although the systems do not always allow for all of the reporting that the commissioners would like.	There are systems in place in providers to collate this data and it is routinely and comprehensively provided to commissioners	

**Ma 3e: Data systems (+ allows sharing): data sharing among local partners**	There is no data sharing between providers and commissioners, or between other agencies.	Some data is shared between commissioners and providers, but not with other agencies.	There is data sharing between providers and commissioners and some data sharing between health and social care.	There is data sharing between providers and commissioners and across agencies, include social care, education and the third sector.	
**Ma 3f: Use of data for performance & QI: Relationships and opportunities for commissioners and providers to jointly consider data**	There are poor relationships between commissioners and providers, making the joint management of contracts difficult.	Although there is performance management of contracts using data, the relationship between the commissioners and providers is not always constructive, making it problematic at times to jointly consider data for managing contracts.	There are good relationships between the commissioners and providers, but there are not always established forums that enable the discussion of this data. While forums are used to support decisions and contracts, it is not utilised as fully to support QI as it could be.	There are opportunities for commissioners and providers to jointly consider performance and quality data for managing contracts and there is a positive, collaborative approach to using this to improve services and inform commissioning.	
**Ma 3g: Use of data for performance & QI: Joint consideration about impact of services**	There are no opportunities between commissioners and providers to jointly discuss about the impact of each service on the whole system's performance.	There have been one or two opportunities in the past between commissioners and providers to consider the impact of services on the whole system, but these are infrequent.	The impact of each provider on the system is frequently considered but that mostly concerns statutory services and does not include the third sector or education.	Data is considered across the full range of providers, with joint consideration of the impact of each service on the whole system's performance.	

THRIVE Principle<br><br>Population level preference data is used to support commissioning decisions	1	2	3	4	Score
**Ma 4b: Collection of preference data**	Data about the preferences of CYP is not regularly collected or looked at by commissioners.	Commissioners may collect preference data through surveys, forums or events, but this is infrequent or ad hoc. This data is unlikely to represent the general population of CYP MH service users (e.g. a small number of CYP is always asked).	Data about CYP preferences is regularly collected from surveys, participation groups, engagement events or other on-population level sources. There is an effort to make sure this data is representative.	Data about CYP's preferences is regularly collected at the population level.	

**Ma 4c: Systems facilitating collection, storage & use**	Providers do not have systems in place to collect and report preference data.	Providers collect and report preference data, but there are no have systems in place to enable systematic data collection.	Providers have systems in place to collect and report preference data, but there are not systems in place to collect quantitative data.	Providers have systems in place to systematically collect and report qualitative and quantitative preference data.	
**Ma 4d: Use of data**	Population level preference data is not used to support commissioning decisions.	Population level preference data is used very infrequently to support (de)commissioning decisions.	There is some evidence that Population level preference data is used to support (de)commissioning in a range of services.	Population level preference data is routinely used to support decision making.	

**THRIVE Principle**<br><br>**Services are working closely together so that service users experience integration of care positively**	**1**	**2**	**3**	**4**	**Score**
**Ma 5b: Measurement of integration of care**	Data is not collected to measure the way services work together.	Some data is collected around the way services work together, but this is infrequent, involves a small sample size or doesn't include all services.	Data is collected around the way services work together is collected by a larger sample size or includes a number of services.	Data is routinely collected around the way services work together.	
**Ma 5c: Systems facilitating collection, storage & use**	There are no systems in place to measure the way services work together.	Services have plans to collect data about the way services work together, but this has not happened yet.	The systems in place to measure the way services work together exist in many services, but this is not across all provider types.	There are systems in place to measure the way services work together across the local service system. This data is shared with commissioners so that they can identify areas of improvement.	
**Ma 5d: Patient experience of integration**	The majority of CYP and families report feeling unhappy with the way services work together.	CYP and families often report feeling unhappy with the way services work together, but there are a few cases where services have worked together well.	CYP and families are mostly satisfied with the way services work together, but there are a few cases where they haven't worked together well.	The majority of CYP report feeling satisfied with the way services work together.	

**MA 5e: CHI ESQ (or other questionnaires relating to service experience)**	CHI ESQ (or other ESQ) are not collected or reported on.	CHI ESQ (or other ESQ) scores are collected, but not systematically.	CHI ESQ (or other ESQ) scores are collected systematically, but this is not reported on.	CHI ESQ (or other ESQ) scores are collected systematically and reported on. (This should recognise alternative systems)	

Collaboration for Leadership in
Applied Health Research and Care
North Thames

i-THRIVE Implementation Assessment Tool Rating Sheet: Meso System Level

THRIVE Principle<br>A comprehensive network of community providers is in place	1	2	3	4	Score
**Me 1a: approach**<br>NB a community provider is an organisation that provides a specific service within the community which is an adjunct to standard NHS care. e.g. bereavement service, peer support groups, parenting support, domestic abuse survivors' group. When working well, the staff in the NHS/LA services will have good relationships with these services and be able to signpost to them after assessment. Ideally commissioners (LA and NHS) will be actively supporting the development of this network through commissioning services.					
**Me 1b - existence of network**	There is no evidence of a network of community providers in place.	The network includes only a few providers, limited to health and social care, or only includes specific commissioned services e.g. a wellbeing service in tier 2.	The network includes a range of providers and includes health, local authority and third sector. There are examples of commissioned and non-commissioned services within the network.	The network includes commissioned and non-commissioned services provided by a range of provider types such as independent (private sector), third sector, local authority, primary care, education. There is evidence that this is working together well.	
**Me 1c - existence of signposting**	There is little effective signposting that is undertaken between the service.	Some effective signposting is undertaken, this is variable and may not happen consistently, or may not take into account the full range of providers.	Signposting regularly takes place to a network of NHS and non-NHS providers locally. Most people in the system will signpost to other providers where appropriate.	The full range of community providers is known about and actively signposted to. There are policies and systems in place to ensure this is systematic and a routine part of service provision.	
**Me 1d - community relationships**	There are no established relationships between community providers and professionals signposting to them.	Community providers have an awareness of each other, however there have not been attempts at an organisational level to build relationships and they currently depend on individual clinicians/professionals/ there is no evidence of relationships existing.	There is an attempt to build relationships with community providers at an organisational level, in particular those that are commissioned, but there is still work to be done in relation to third sector/other independent organisations.	There are good relationships with community providers from all sectors, with evidence of joint working and collaboration in the delivery of care.	

**Me 1e - understanding of referral procedures between providers**	There is no understanding of referral procedures between providers. No training is provided by organisations regarding referral procedures and rejections are not accompanied by explanations.	There is no detailed consideration of referral processes to community providers. Or this is patchy with no systematic consideration across services.	There is a reasonable understanding of the criteria for entry and referral processes of the providers most often signposted to. These are generally agreed and adhered to.	Criteria for entry and referral processes to community providers are known by all professionals that are signposting and they are mostly adhered to.	
**Me 1f - access to (up to date) network information**	Knowledge of network information is dependent on the individual clinician as there has been no formal attempt to collate this. There is no way for CYP or their families to access information about services locally.<br><br>Signposting may happen effectively in isolated cases but there are no effective systems in place to enable this to happen routinely as part of assessment or ongoing care planning.	There is an attempt to collate information about the range of services available, but this is not comprehensive, may sit in a number of different places. Practitioners do not routinely use it to help CYP understand their options. CYP and their families are not currently able to access this information easily. Much of the information is out of date.	Information about community providers is maintained in a single place, although this may be difficult for CYP to use or access. It is not clear how information about community providers is kept up to date.	There is a single digitally enabled database of the full range of community services available that is maintained regularly (e.g. Youth Wellbeing Directory). CYP are able to access information to be able to support them to access these services themselves.	
**Me 1g - effectiveness & re-referrals**	Substantial numbers of referrals are sent back with no other more suitable option for care to be identified or referred to. There is evidence that the community provision is underused and CAMHS providers feel they are being asked to manage a large number of cases that ‘don’t meet their criteria’, or these cases are refused care with no alternatives as they ‘don’t meet the threshold for CAMHS’.	There is some evidence of CYP re-presenting for CAMHS assessment due to ineffective signposting, or frustration from community providers due to a high number of inappropriate referrals to them.	There is some evidence that signposting is working effectively – referrers do not have their referrals returned without advice on what services are helpful, and community providers are not overwhelmed with inappropriate referrals due to lack of understanding of their service criteria.	Community providers feel that most referrals into their services are appropriate as a result of effective signposting from the NHS. There are low number of re-presentations to CAMHS as a result of failed signposting.	

THRIVE Principle<br><br>Quality improvement data is used to inform decisions, and this includes multi-agency considerations of the data using the 'CORC Mindful Approach'	1	2	3	4	Score
**The 'MINDFUL approach' is a CORC mindfulness measure. The following elements of the measure are relevant to the meso system and would ideally be present in a THRIVE-like system:**<br>**1.** Outcome and process measures are collected routinely and used to help shape service provision.<br>**2.** There is an outcomes framework that addresses all the THRIVE-groups.<br>**3.** Data is collated and feedback to staff to support QI work.<br>**4.** There are systems in place that enable staff to discuss and explore variations in quality data.<br>**5.** QI projects are undertaken using recognised methodology (e.g. PDSA) to reduce variation and improve quality of services.<br>**6.** There are regularly (e.g. quarterly) learning forums in place that involve all sectors.<br>**7.** There is an annual review of services that is undertaken					
**Me 2b: routine collection of outcome & process measures**	Very few outcome & process data are collected. Data collection is not systematic across any providers.	Outcome & process data are collected by some providers, however this is not done systematically.	Outcome & process data are collected by most providers. There are some efforts to collect these systematically.	A comprehensive set of outcome and process data are collected. Data collection is widespread across all providers and undertaken systematically.	
**Me 2c: outcomes framework according to 5 THRIVE groups**	There is no outcome framework used for data collection.	There is an outcome framework, but it does not address the 5 THRIVE groups.	There is an outcome framework where some of THRIVE groups are implicitly addressed, but not explicitly.	There is an outcomes framework that addresses all the 5 THRIVE groups	
**Me 2d: data is fed back to staff**	Outcome data is rarely fed back to staff.	Outcome data is sometimes fed back to staff, however it is not collated or explained making it difficult to embed	Outcome data is often collated and fed back to staff.	Outcome data is collated and is systematically fed back to staff to use in practice.	

**Me 2e: systems to discuss data**	There are no systems in place to enable the discussion of QI data.	There are some systems in place to enable discussions around QI data. Discussions around data may happen in an ad hoc way.	There are systems in place which are used to discuss QI data. Those are used often but not systematically.	There are systems in place that enable discussions around data. Those systems are used routinely to explore variations in QI data.	
**Me 2f: QI projects**	There are no QI projects undertaken to improve service quality.	There are some QI projects undertaken to improve service quality, but this happens rarely.	QI projects are undertaken to improve quality of services, but this is not systematic.	QI projects are undertaken routinely using recognised methodology (e.g. PDSA cycles) to reduce variation and improve quality of services.	
**Me 2g: learning forums**	There are no learning forums in place which involve other sectors.	There are learning forums in place, however these are sporadic and only involve two agencies (e.g. only health and social care).	There are regular learning forums in place (e.g. quarterly). These have multi agency input however do not involve all sectors (e.g. health, social care, education, third sector).	There are regular learning forums in place (e.g. quarterly) that involve all sectors, including non-commissioned services.	
**Me 2h: annual review**	There is no annual review of services undertaken.	There is an annual review of services undertaken by one or two agencies agency in the locality.	There is an annual review of services undertaken by most agencies in the locality.	There is an annual review of all services' reviews which involves most agencies in the locality. A single report brings these together.	

THRIVE Principle<br><br>**Help is delivered using the THRIVE conceptual framework of five needs-based groups**	1	2	3	4	Score
**Me 3b: grouping is multiagency**	There are no plans for grouping according to the THRIVE conceptual framework.	The implementation has not fully involved all agencies at this stage, although plans are in place to enable this.	Implementation includes all agencies but it may not be fully mature in all of them.	Implementation includes all agencies.	
**Me 3c: service delivery**	None or only one of the THRIVE needs based groups are fully implemented.	Two THRIVE needs based groups are fully implemented.	Three or four of the THRIVE needs based groups are fully implemented.	All of five of the THRIVE groups are fully implemented. Services are organised to deliver needs-based care according to the five groups.	

**Me 3d: assessment according to THRIVE Groups**	There are plans to assess CYP needs according to THRIVE principles but implementation is not yet underway, or has not translated to clinical practice yet.	CYP needs are assessed according to the THRIVE Framework but services are not yet set up to clearly align to each needs based group.	Assessment according to Need is established, CYP are allocated to a THRIVE group, and CYP are able to access care according to the THRIVE needs based groups as a result of that assessment.	There is an effective assessment process that enables CYP to be signposted into the right THRIVE needs based group for them as part of a shared decision.	
**Me 3e: grouping systems for recording**	There are no systems in place to report on activity or outcomes for each THRIVE needs based group.	Reporting according to THRIVE needs based group is being planned, but has not been implemented.	Some reporting systems are in place and some services are able to report based on THRIVE group.	There is a way of recording THRIVE needs based groups in electronic patient records. It is possible to report on the activity and outcomes for each of the THRIVE needs based groups.	
**Me 3f: Staff survey**	There is no staff survey (asking about THRIVE conceptual Framework's needs based groups) across any providers.	There is a staff survey (asking about THRIVE conceptual Framework's needs based groups) undertaken by at least one agency in the locality.	There is a staff survey (asking about THRIVE conceptual Framework's needs based groups) undertaken by 2+ agencies in the locality.	There is a staff survey (asking about THRIVE conceptual Framework's needs based groups) undertaken by all agencies in the locality.	

THRIVE Principle<br><br>**There is a focus on the strengths and resources of the family wherever possible**	1	2	3	4	Score
**Me 4a: approach & consideration of the strengths and resources of the family**	There is no consistent consideration of the family and the wider support network.	There is some consideration of the family and the wider support network. This takes place in one sector.	There is consideration of the family and the wider support network most of the time. This takes place across 2-3 sectors.	There is evidence that consideration of family and the wider support network almost always happens. This takes place across all sectors.	
**Me 4b - resources of the family are considered in care plans**	Resources of the family are not included in care plans. This is not routinely documented therefore it would not be possible to audit this information.	Resources of the family are sometimes included in care plans, but documentation is not consistent. It would not be possible to reliably audit this currently.	Resources of the family are included in care plans in the majority of cases. Documentation is of good quality. It would be possible to reliably audit this currently.	Resources of the family are considered systematically in the development of care plans. This is routinely documented and can be audited.	

**Me 4c - patient activation & self-management**	Self-management and patient activation are not an explicit part of the approach to supporting CYP.	Self-management and patient activation are sometimes part of the approach to supporting CYP.	Self-management and patient activation are often an explicit part of the approach to supporting CYP.	Self-management and patient activation is actively promoted and supported. Clinicians are able to support CYP with this.	
**Me 4d - training**	There is no opportunity for practitioners to develop these skills currently.	There has been at least one training for practitioners to develop these skills. Overall few practitioners have received this training.	There have been several trainings available to develop these skills, however not all practitioners have the opportunity to develop these skills currently.	Training programmes that support practitioners in this are openly available and most have attended.	

THRIVE Principle<br>**Evidence based practice is available and aligned to need using the 19 sub-categories of needs set out in the payment systems work**	1	2	3	4	Score
**Me 5b: EBP availability**	Evidence based practice is only provided by very few services and provision may be inconsistent within services.	Evidence based practice is available in some services. It may be inconsistently provided within services.	Evidence based practice is available in most services but not all.	Evidence based practice is routinely available in all services.	
**Me 5c: EBP accessibility**	Most CYP are not able to access evidence-based interventions such as therapies,	Some CYP can access evidence-based practice.	Most CYP can access evidence-based practice.	All CYP are able to access evidence-based practice, for a wide range of conditions.	
**Me 5d: EBP 19 categories**	Evidence based practice is not aligned to the 19 categories of need.	Some evidence-based practice is aligned to the 19 categories of need.	Most evidence-based practice is aligned to the 19 categories of need.	Evidence based practice is aligned to the 19 categories of need.	

**Me 5e: EBP systems to quantify access**	It is not possible to quantify how many CYP have access to evidence-based practice, there are no systems in place to do this.	It would be very difficult to quantify how many CYP have access to evidence-based practice as systems are not designed to collect this information.	It would be possible to quantify how many CYP receive evidence-based practice (e.g. by looking at access to certain services or therapists), however systems were not designed to collect this information.	There are systems in place to quantify the number of CYP able to access evidence-based practice. This data would not be difficult to access.	
**Me 5f: NICE guidelines**	Evidence of alignment of therapy with NICE-based guidance, where clearly relevant (e.g. CBT for anxiety) is achieved for at least 20% of relevant cases.	Evidence of alignment of therapy with NICE-based guidance, where clearly relevant (e.g., CBT for anxiety) is achieved for at least 40% of relevant cases.	Evidence of alignment of therapy with NICE-based guidance, where clearly relevant (e.g., CBT for anxiety) is achieved for at least 60% of relevant cases.	Evidence of alignment of therapy with NICE-based guidance, where clearly relevant (e.g., CBT for anxiety) is achieved for at least 80% of relevant cases.	

i-THRIVE Implementation Assessment Tool Rating Sheet: Micro System Level

THRIVE Principle<br><br>Shared decision making is in the heart of all decisions	1	2	3	4	Score
**Mi 1a: Approach**<br>SDM at a micro level is when a decision about a care is made collaboratively between the professional and the service user (and family). A collaborative conversation between the service user and professional providing care takes place in order to decide on the best management for the service user. Their views are taken into account in these decisions. Ideally this approach would be taken wherever the patient receives care (NHS, LA, education or third sector). Patient reported data on shared decision making is critical to this.					
**Mi 1b: SDM is multi-agency**	Shared decision making has not been implemented in any agency.	Shared decision-making is occasionally used in practice in some agencies.	Shared decision-making is part of clinical practice, sometimes other agencies are also involved but this will be limited to either health or social care.	Shared decision-making is part of clinical practice, other agencies are often involved to assist service users to make informed decisions	
**Mi 1c: training**	There is no SDM training available to staff.	There has been at least one training for practitioners to develop these skills. Overall few practitioners have received this training.	There have been several trainings available to develop these skills, however not all practitioners have the opportunity to develop these skills currently.	Training on SDM is openly available and the majority of staff have attended.	
**Mi 1d: measurement of SDM**	No measures of SDM have been implemented in any services.	CollaboRATE or another measure of SDM has been implemented in one setting (health, social care, third sector, education).	CollaboRATE or another measure of SDM has been implemented in two or three settings (health, social care, third sector, education).	CollaboRATE or another measure of SDM has been implemented across all settings (health, social care, third sector, education).	
**Mi 1e: systems for collection, use & reporting**	There are no systems in place to collect and report data regarding decision making.	Data on decision making is collected, but this is occasional, and data is not reported.	There are some systems in place to collect data on decision making and it is often reported back to staff.	There are systems in place to collect data on decision making. Data is routinely reported back to staff in a format that facilitates quality improvement work.	
**Mi 1f: articulated in strategy**	SDM is not articulated in the organisation's strategy.	SDM is articulated in the organisations' strategy however is not regarded as a priority.	SDM is articulated in the strategy and is regarded as a priority.	SDM is clearly articulated in the strategy, is regarded as a priority and there are clear steps articulated to promote SDM.	

Collaboration for Leadership in Applied Health Research and Care North Thames

THRIVE Principle<br><br>**People are clear about which needs based group they are working within for any one person at any one time and this is explicit to all (professionals, CYP and families)**	1	2	3	4	Score
**Mi 2b: CYP are categorised according to need**	CYP are categorised by diagnosis, severity or another way that is not needs-based.	CYP may be described by their needs, but this is not routine and does not form the basis of service provision.	CYP are categorised based on their needs in some cases.	CYP are categorised by distinct needs-based groups (eg. THRIVE groups).	
**Mi 2c: awareness of group – professionals**	Professionals are not aware of the needs group of the CYP they support.	Professionals are sometimes aware of the needs group of the CYP they support.	Most professionals are aware of the needs group of the CYP they support.	Professionals are always aware of the needs group of CYP they support. This is documented routinely in care plans or case notes.	
**Mi 2d: awareness of group - CYP**	CYP are not aware of the needs based group they are in.	CYP may be passively told about their needs based group (e.g. through a letter) but this is not discussed in person.	There is usually an in-person discussion with CYP about their needs based group, but this is not done systematically and may vary between clinicians.	CYP are routinely part of an explicit discussion about the different needs based groups and are aware of how they have been categorised.	
**Mi 2e (i): recording of group**	20% of notes have the needs based group recorded.	40% of notes have the needs based group recorded.	60% of notes have the needs based group recorded.	80% of notes have the needs based group recorded.	
**Mi2e (ii): explicit discussion recorded**	20% or less notes show an explicit discussion between professional and CYP about the most suitable needs-based group.	40% or less notes show an explicit discussion between professional and CYP about the most suitable needs-based group.	60% or less notes show an explicit discussion between professional and CYP about the most suitable needs-based group.	80% or less notes show an explicit discussion between professional and CYP about the most suitable needs-based group.	

THRIVE Principle<br><br>**People are clear about parameters for help and reasons for ending treatment (professionals, CYP and families)**	1	2	3	4	Score
**Mi 3b: training on ending treatment**	Staff do not have access to training on when to end treatment.	There has been at least one training for practitioners to develop these skills. Overall few practitioners have received this training.	There have been several trainings available to develop these skills, however not all practitioners have the opportunity to develop these skills currently.	Most staff have access to training on when to end treatment.	
**Mi 3c - reasons & parameters discussed at beginning (+ staff awareness)**	Reasons for ending are never, or rarely, addressed at the beginning of therapy.	Reasons for ending are sometimes addressed at the beginning of therapy, however this is not routine and may vary by clinician.	Reasons for ending are often addressed at the beginning of therapy, however this is not routine and may vary by clinician.	Reasons for ending are routinely addressed at the beginning of therapy.	
**Mi 3d - staff understand importance of endings**	Staff do not recognise that discussing reasons for ending is an important part of all therapy sessions.	Some staff recognise that discussing reasons for ending an important part of all therapy sessions.	Most staff recognise that discussing reasons for ending is an important part of all therapy sessions.	Nearly all staff recognise that discussing reasons for ending is an important part of all therapy sessions.	
**3e: Reasons for ending proforma – reasons documentation which should document the conversations had at the beginning of therapy**	Reasons for ending are not routinely documented. There are no systems in place to facilitate this.	There are no systems in place to document reasons for ending treatment however, this may be documented in some clinicians notes but this is not routine.	There are systems in place to document reasons for ending and this may be documented but this is not routine.	There are systems in place to document reasons for ending. This is documented most of the time.	
**3f: Reasons for ending proforma**	0-40% of case notes have the reasons for ending proforma filled out and this confirms that there was explicit consideration of endings and that this was discussed with CYP and their families at the beginning of therapy.	40% - 59% of case notes have the reasons for ending proforma filled out and this confirms that there was explicit consideration of endings and that this was discussed with CYP and their families at the beginning of therapy.	60-79% of case notes have the reasons for ending proforma filled out and this confirms that there was explicit consideration of endings and that this was discussed with CYP and their families at the beginning of therapy.	80-100% of case notes have the reasons for ending proforma filled out and this confirms that there was explicit consideration of endings and that this was discussed with CYP and their families at the beginning of therapy.	
**Mi 3h - CYP understand parameters & reasons**	Children and young people do not have a clear understanding about the parameters for help available and the reasons for ending treatment.	Only a few children and young people have a clear understanding about the parameters for help available and the reasons for ending treatment.	Most of children and young people have a clear understanding about the parameters for help available and the reasons for ending treatment.	Nearly all children and young people have a clear understanding about the parameters for help available and the reasons for ending treatment.	

THRIVE Principle<br><br>Outcome data is used with the purpose of improving quality	1	2	3	4	
**Mi 4b: collection & utilisation of outcome data**	Routine outcome data is not collected, and it is not used as part of QI processes within services.	Routine outcome data is collected and used to support QI processes in some services.	Routine outcome data is collected and utilised to support QI processes for most services.	Routine outcome data is collected and utilised to support QI processes within all services.	
**Mi 4c: systematic collection of outcome data**	There are no systems in place to enable the collection of routine outcome data.	There are systems in place to enable data collection found in the services of one sector in the locality.	There are systems in place to enable data collection found in the services of two or three sectors in the locality.	There are systems in place to enable the collection of routine outcome data across all services.	
**Mi 4d: outcomes strategy**	Collection of routine outcome data is not routinely done nor is it an explicit part of the organisational strategy.	Collection of routine outcome data does happen in isolated areas but it is not yet a part of the organisation's culture.	Collection of routine outcome data takes place in most areas and is used to inform individual practice but this is not standardised across services.	Collecting routine outcome data is part of the organisational strategy and there are specific times and places (e.g. a team meeting, or during supervision) where outcomes and any variations in outcomes between teams or individuals are discussed.	
**Mi 4e - QI culture**	QI is not a part of the culture in service provision	QI is actively promoted across local services but only within services of one sector (health, social care, education, third sector).	QI is actively promoted across local services but only within services of two or three sectors (health, social care, education, third sector).	QI is a strong part of the culture within local service provision, including all sectors.	
**Mi 4f - QI training**	Almost no staff have had QI training.	Some staff have had QI training.	A lot of staff are familiar with QI approaches and have some experience in the use of a standardised QI methodology.	Almost all staff are familiar with QI approaches and feel confident in the use of a standardised QI methodology.	

THRIVE Principle<br><br>Intervention involves explicit agreement about the goal being worked towards and the likely time frame. There is a plan for what happens if this is not achieved.	1	2	3	4	Score
**Mi 5b: goals discussion**	Goals and expected outcomes are not discussed at the beginning of treatment.	Goals and expected outcomes are sometimes discussed at the beginning of treatment, but this varies case to case.	Goals and expected outcomes are often discussed and agreed with CYP and families at the beginning of treatment.	Goals and expected outcomes are discussed and agreed with CYP and families at the beginning of treatment most of the time.	
**Mi 5c: goals recorded**	Goals are not recorded in case notes.<br><br>Case audit: in 0-39% of notes, the goals and expected outcomes for treatment are discussed with CYP and their families and recorded in notes.	Goals are sometimes recorded in case notes.<br><br>Case audit: in 40-59% of notes, the goals and expected outcomes for treatment are discussed with CYP and their families and recorded in notes.	Goals are often recorded in case notes.<br><br>Case audit: in 60-79% of notes, the goals and expected outcomes for treatment are discussed with CYP and their families and recorded in notes.	Goals are systematically recorded in case notes.<br><br>Case audit: in 80-100% of notes, the goals and expected outcomes for treatment are discussed with CYP and their families and recorded in notes.	
**Mi 5d: plan when goals not achieved**	There is very rarely a discussion about what happens if the goal is not achieved.	There is sometimes a discussion round what happens if the goal is not achieved, but this varies by client and practitioner.	There is often a discussion around what happens if the goal is not achieved, but this may not result in a plan.	There is always a discussion around what happens if a goal is not achieved and a plan is then put in place.	
**Mi 5e: plan recorded when goals not achieved**	If there is a discussion about what happens if a goal Is not achieved no plan is recorded in case notes most of the time.	The plan for what happens if a goal Is not achieved is not usually recorded in case notes.	The plan for what happens if a goal Is not achieved is often included in case notes.	The plan for what happens if a goal Is not achieved is systematically recorded and regularly reviewed.	
**Mi 5f: sessions (e.g. NICE)**	CYP are usually not managed within the NICE recommended number of sessions.<br><br>Case audit: 0-39% CYP and their families are managed within the recommended number of therapy sessions according to NICE guidelines.	Less than half of CYP are managed within NICE recommended number of sessions.<br><br>Case audit: 40-59% CYP and their families are managed within the recommended number of therapy sessions according to NICE guidelines.	Many CYP are managed within the NICE recommended number of sessions.<br><br>Case audit: 60-79% CYP and their families are managed within the recommended number of therapy sessions according to NICE guidelines.	Nearly all CYP are managed within the NICE recommended number of sessions.<br><br>Case audit: 80-100% CYP and their families are managed within the recommended number of therapy sessions according NICE guidelines.	

THRIVE Principle<br><br>The most experienced practitioners inform advice and signposting	1	2	3	4	Score
**Mi 6a: approach to supervision of advice & signposting**	There is not a Grade 8 or above mental health practitioner involved in the signposting and advice provided across the services.<br><br>There is no way for these teams to access supervision by someone with mental health expertise.	There is at least one Grade 8 or above mental health practitioner involved in giving advice and supporting signposting.<br><br>Some of the assessments in the community (e.g. local authority teams or schools) are not routinely discussed with a senior mental health practitioner.	There is at least one Grade 8 or above mental health practitioner involved in giving advice and supporting signposting.<br><br>Most individuals across the system feel able to get support from the senior clinician when they are providing advice and signposting to CYP and their families.	There is at least one Grade 8 or above mental health practitioner involved in giving advice and supporting signposting.<br><br>All individuals across the system feel able to get support from the senior clinician when they are providing advice and signposting to CYP and their families.	
**6b: signposting is multiagency**	There are multiple teams in different settings that provide advice and signposting. The approach is not co-ordinated across the system.	There are distinct advice and signposting services, but these are primarily staffed and used by one agency (eg. CAMHS). Individuals from other agencies are not linked into a centralised hub.	Most individuals across the system are linked into the advice and signposting services.	The team undertaking assessments is multi-disciplinary and/or multi-agency but acts as a coherent team, signposting systematically throughout the system.	
**6c: systems to enable supervision of signposting**	There are no systems in place through which the senior mental health practitioner can be assured that the advice and signposting systems are operating effectively.	There are some systems in place through which the senior mental health practitioner can be assured that the advice and signposting systems are operating effectively, however these are not used frequently and do not provide adequate detail.	There are systems in place through which the senior mental health practitioner can be assured that the advice and signposting systems are operating effectively, these are used fairly frequently.	There are effective systems in place through which the senior mental health practitioner can be assured that the advice and signposting systems are operating effectively. These are used systematically.	
**Mi 6d: signposting effectiveness is measurable**	There is no way to measure how effective the advice and signposting across the system works.	There is some effort to measure frequent re-referrals or effective signposting but this is not done systematically.	There is are some specific measures in place to measure re-referrals or effective signposting and they are used some of the time.	There are systems in place which routinely measure frequent re-referrals or effective signposting and they are used most of the time.	

Collaboration for Leadership in Applied Health Research and Care North Thames

THRIVE Principle<br><br>**THRIVE plans are used to help those managing risk**	1	2	3	4	Score
**Mi 7b: risk plan is multiagency**	Risk plans are not developed alongside or shared with other agencies.	Risk plans may be developed alongside and shared with other agencies, but this is rare or happens only upon request.	Risk plans are developed alongside other agencies in some cases, but not all.	There is an agreed multi-agency plan for CYP receiving risk support. All agencies involved are signed up to the plan.	
**Mi 7c: risk plan updated regularly**	Risk plans are often not kept up to date.	Risk plans are sometimes kept up to date.	Risk plans are mostly kept up to date.	Risk plans are routinely updated and audited regularly.	
**Mi 7d: recording of risk plan**	Case audit: 0-39% of CYP in the ‘Getting Risk Support’ needs based group have a THRIVE plan documented and up to date.	Case audit: 40-69% of CYP in the ‘Getting Risk Support’ needs based group have a THRIVE plan documented and up to date.	Case audit: 60-79% of CYP in the ‘Getting Risk Support’ needs based group have a THRIVE plan documented and up to date.	Case audit: 80-100% of CYP in the ‘Getting Risk Support’ needs based group have a THRIVE plan documented and up to date.	
