# Supplementary material for: A protocol for a multi-site cohort study to evaluate child and adolescent mental health service transformation in England using the i-THRIVE model
Source: PLoS One. 2023 May 8;18(5):e0265782. doi: 10.1371/journal.pone.0265782 (PMC10166497; doi:10.1371/journal.pone.0265782)
Supplement: Attachment — Submitted filename: Response to Reviewers1.docx [file pone.0265782.s007.docx]

Tel: +44 (0) 1223 339396

Fax: +44 (0) 1223 764062

Email: am2708@medschl.cam.ac.uk

www.cam.ac.uk

**Journal Requirements:**

When submitting your revision, we need you to address these additional requirements.

1. Please ensure that your manuscript meets PLOS ONE's style requirements, including those for file naming. The PLOS ONE style templates can be found at

<https://journals.plos.org/plosone/s/file?id=wjVg/PLOSOne_formatting_sample_main_body.pdf>  and

<https://journals.plos.org/plosone/s/file?id=ba62/PLOSOne_formatting_sample_title_authors_affiliations.pdf>

We have amended the formatting to be in line with the guidelines.

2. We note that you have stated that you will provide repository information for your data at acceptance. Should your manuscript be accepted for publication, we will hold it until you provide the relevant accession numbers or DOIs necessary to access your data. If you wish to make changes to your Data Availability statement, please describe these changes in your cover letter and we will update your Data Availability statement to reflect the information you provide.

We intended to say that the data would not be available until the end of the study (which will be after this protocol is published). This may not have been clear enough in our section on Data Access and so we have re-drafted this to provide additional clarity.

3. Please review your reference list to ensure that it is complete and correct. If you have cited papers that have been retracted, please include the rationale for doing so in the manuscript text, or remove these references and replace them with relevant current references. Any changes to the reference list should be mentioned in the rebuttal letter that accompanies your revised manuscript. If you need to cite a retracted article, indicate the article’s retracted status in the References list and also include a citation and full reference for the retraction notice.

We have refreshed the reference list and as far as we are aware, we have not cited any papers that have been retracted.

**Responses to reviewers:**

**Reviewer #1:** The study protocol has hugely ambitious scope and has the potential to directly influence services for CYP mental health and also how implementation science is undertaken.

Thank you for this supportive recognition of the scope and ambition we have for this project.

Major queries:
How were/will the THRIVE intervention and non-THRIVE control sites be selected? Understanding what might drive differences in THRIVE/control sites at baseline is central to the evaluation and is not sufficiently well described.
The sites have been selected. We have included additional information about the process of selection for both controls and test sites (see section ‘study design and setting’).

There are two key considerations; i) how you select the comparison group, ii) how you understand what was implemented in place of THRIVE. Both are critical for the evaluation. If the control sites have not yet been selected then it would be helpful to describe the data that will be used to identify sites with similar characteristics (size, urbanicity, deprivation etc). If these are available at national level then please consider running several comparison approaches in parallel for at least some aspects of the evaluation – for example using synthetic control groups that have similar patterns of service use before the start of the study.

We agree that these are both critical points – thank you for highlighting these. We have aimed to provide clarification on our approach in response to this. We have provided detail about the approach to selection of the comparison group in the section ‘study design and setting’. We recognise the need to match the sites as closely as possible in terms of baseline characteristics, as well as commitment to transformation and approach to joint working. To address this, we matched initially on baseline characteristics, and then interviewed preferred sites to find sites motivated to transform CAMHS and take a partnership approach between health and social care. We also required them to be willing to participate in the evaluation for four years and to commit not to implement i-THRIVE within this time period. Part of our initial analysis will be to measure the fidelity to THRIVE at baseline. We will then be able to determine the extent to which this changes over the implementation period. We will report this in the first results paper.

Minor points:
Lines 87/88: given how widespread the coverage of the THRIVE intervention is reported to be, can you add some further information about how sites opted in (or out) of this model.

This detail has been added.

Lines 92-126: I agree that place-based care is increasingly important (e.g. given the shift towards integrated care systems), however, I felt that it wasn’t clear which services were viewed as being part of this system. Because of this it is hard to gauge whether the outcomes of interest have wide enough breadth – e.g. knock effects on CYP mental health presentations to A&E. Some more information about which services this relates to would be helpful either here or in the section on Service Transformation within CAMHS.

We have included detail of how we understand integrated care systems for the purpose of this study.

Lines 230-241: I am unclear how data collection works at sites where THRIVE has not been implemented.

This section describes the approach to implementation in i-THRIVE sites rather than the data collection approach. We collected data in the same way in both control and test sites: interviews, surveys and use of de-identified routinely collected CAMHS data. The same processes and approach was used in all sites.

Table 2: not entirely clear how some of these will be measured and compared – e.g. clinical outcomes mean baseline need/severity, as measured by site.

At the moment we are not able to set this out, as we have not been able to collect the data from all sites. Most sites use CGAS and so we expect this to be the most likely outcome measure that will be used. We will be able to develop the measurement approach in detail once the data has been collected and mapped across the sites.

Line 350-398: is it likely that some control sites may be quite “THRIVE-like”? Will this change what you do if this is the case?

We don’t think so. We hope that at base line the controls and test sites are similarly THRIVE-like, as this will be a good indicator that the matching process was successful. Our assessment will be to determine the change in THRIVE-likeness over the 4 years. If the controls are equally as THRIVE-like, then we are likely to conclude that CAMHS transformation as driven by NHS England leads to similar CAMHS service structure changes regardless of the model and approach to implementation taken. We hypothesise that sites participating in the i-THRIVE Implementation programme will be more THRIVE-like at follow up. And we will then test the relationship between THRIVE-likeness and outcomes.

Line 416-425: please consider using a wider range of comparison groups and methods for the quantitative outcomes (if possible)

We have significantly expanded the analysis section by including a more detailed description of our plans.

**Reviewer #2:** This is a very thorough protocol for an ambitious and impressive study. The only section that I thought may benefit from more detail (or reference to more detail reported elsewhere if applicable) was the planned data analysis section.

Thank you for the comments on the protocol. We have significantly expanded the analysis section by including a more detailed description of our plans.
