# Supplementary material for: A protocol for a multi-site cohort study to evaluate child and adolescent mental health service transformation in England using the i-THRIVE model
Source: PLoS One. 2023 May 8;18(5):e0265782. doi: 10.1371/journal.pone.0265782 (PMC10166497; doi:10.1371/journal.pone.0265782)
Supplement: Attachment — Submitted filename: Response to reviewers.docx [file pone.0265782.s008.docx]

| Dr Anna Moore  NIHR Anna Freud Clinical Lecturer in Child Psychiatry |
| --- |
|  |

01.03.2023

Dear Dr. Werdhani

**REF: PLOS ONE Decision: Revision required [PONE-D-22-03202R1] - [EMID:c1b1b73285a936f7]**

Thank you for your sharing the editors and reviewers’ comments with us. Please find enclosed

1. A rebuttal letter that responds to each point raised by the academic editor and reviewer(s).
2. A marked-up copy of your manuscript that highlights changes made to the original version.
3. An unmarked version of your revised paper without tracked changes.

Journal Requirements:

1. **Please review your reference list to ensure that it is complete and correct. If you have cited papers that have been retracted, please include the rationale for doing so in the manuscript text, or remove these references and replace them with relevant current references. Any changes to the reference list should be mentioned in the rebuttal letter that accompanies your revised manuscript. If you need to cite a retracted article, indicate the article’s retracted status in the References list and also include a citation and full reference for the retraction notice.**

We have reviewed the reference list and all 83 references have been checked and updated as required.

Additional Editor Comments:

2. **Need to be have the definition of urbanicity, funding, level of deprivation and expected prevalence mental health care needs for matching variables**

We thank the Editor for these comments and have attempted to comply with each. In relation to definitions of matching criteria we have referred to the ONS 2015 Survey which we used as the bases. The following text has been added to the study setting and design section of the manuscript on page 17:

*Our approach was to match for baseline characteristics first (population size and density reported by the Office of National Statistics – Mid 2015 Population Estimates for Clinical Commissioning Groups in England, combined CAMHS and CCG funding 2016-2017 and level of deprivation defined by the English Indices of Deprivation 2015.*

Reviewers' comments:

3. **The calculation of N = 10 for each case and control
   The method is not case control. It’s more like a cohort (start with the exposure vs non exposure: I thrive is considered the exposure, and then will be followed up until 4 years after service implementation. If you used secondary data, then the method will be historical cohort.**

Thank you for your feedback.

We have updated the description of the study design to cohort study. The justification of the number of case and control sites chosen was based on the number of early adopters identified which could be reasonably matched with other sites using different approaches to service transformation that were comparable in size and demographics. The number is also consistent with minimum size of a cluster randomised control trial design (Medical Research Council (2002) Cluster Randomised Trials: Methodological and Ethical Considerations. MRC, London. Available at <http://www.mrc.ac.uk/index/publications/pdf->) Cluster trials with fewer than five clusters per arm are considered inadvisable. We attempted to balance out cluster level confounding by matching on key demographics. As the study is a cohort study there are only pragmatic rather than statistical justification for the number of sites selected.

4. **The** [**PLOS Data policy**](https://eur01.safelinks.protection.outlook.com/?url=https%3A%2F%2Fjournals.plos.org%2Fplosone%2Fs%2Fmaterials-and-software-sharing&data=05%7C01%7Cp.fonagy%40ucl.ac.uk%7C42476b36373448c384fc08da9a3a9ff0%7C1faf88fea9984c5b93c9210a11d9a5c2%7C0%7C0%7C637991874420665309%7CUnknown%7CTWFpbGZsb3d8eyJWIjoiMC4wLjAwMDAiLCJQIjoiV2luMzIiLCJBTiI6Ik1haWwiLCJXVCI6Mn0%3D%7C3000%7C%7C%7C&sdata=RAOyAgzJMp2tUkaMasouSsVqHh7e2X5dC2zEwrQJ8cg%3D&reserved=0) **requires authors to make all data underlying the findings described in their manuscript fully available without restriction, with rare exception, at the time of publication. The data should be provided as part of the manuscript or its supporting information, or deposited to a public repository. For example, in addition to summary statistics, the data points behind means, medians and variance measures should be available. If there are restrictions on publicly sharing data—e.g. participant privacy or use of data from a third party—those must be specified.**

Our understanding is that because the manuscript is a protocol the requirement to make data available does not apply.

We are grateful for the opportunity we have been given to improve our manuscript and we have done our best to address all the issues raised. We will be delighted to make any further amendments you feel may be required. We look forward to hearing from you in due course.

Warm regards,
